# Abdominal Sepsis With Pseudomonas aeruginosa After Oocyte Retrieval

**DOI:** 10.7759/cureus.82149

**Published:** 2025-04-12

**Authors:** Vashisht V Persad, Nigel Bascombe

**Affiliations:** 1 Obstetrics and Gynecology, Sanjivani Women's Hospital, St. Augustine, TTO; 2 General and Laparoscopic Surgery, Sanjivani Women's Hospital, St. Augustine, TTO

**Keywords:** abdominal sepsis, assisted reproductive technology (art), egg retrieval, in-vitro fertilization (ivf), oocyte retrieval, pseudomonas aeruginosa

## Abstract

We present a case of a 30-year-old female admitted to our hospital with signs of intra-abdominal sepsis, without a clear source, after undergoing controlled ovarian stimulation and egg retrieval. She failed to improve after 48 hours of broad-spectrum antibiotic therapy and subsequently underwent diagnostic laparoscopy. Intra-abdominal findings included diffuse pus and fibrinous exudates, without abscess formation. Peritoneal washout and prophylactic appendectomy were performed, with dramatic improvement post-operatively. Cultures of intra-abdominal pus grew *Pseudomonas aeruginosa*, and pathology confirmed that the appendix was not involved. Given that the rectouterine pouch appeared to be the most inflamed area and given the lack of other exposures, our case demonstrates a severe intra-abdominal infection following oocyte retrieval during in-vitro fertilization treatment. This is a rare, serious complication, and physicians should maintain a high index of suspicion for this condition in patients with similar presentation, as early diagnosis and intervention are crucial.

## Introduction

Worldwide, the use of assisted reproductive technologies (ART) is increasing, with a growing number of oocyte (egg) retrievals being performed for both infertility treatment [1] and oocyte cryopreservation [2]. In general, oocyte retrieval is considered a low-risk procedure, with major risks being intra-abdominal bleeding and ovarian hyperstimulation syndrome. Infection is rare, with a cited incidence of one in 500, but is a severe and potentially deadly complication. Infection usually is secondary to seeding with vaginal flora. Although *Pseudomonas aeruginosa* is occasionally found in healthy vaginal flora, this is, to our knowledge, the first reported case of abdominal sepsis caused by this organism following egg retrieval. While the overall risk of infection is low, our case highlights the importance of maintaining vigilance for this diagnosis in patients presenting with abdominal pain or other unusual symptoms after the procedure.

## Case presentation

Our patient was a 30-year-old female nulligravida with no significant medical history who presented with worsening diffuse abdominal pain over the past few days. She also reported occasional low-grade fevers and chills at home prior to admission and associated nausea and decreased appetite. She had been undergoing assisted reproductive treatments due to male factor infertility and underwent transvaginal oocyte retrieval under ultrasound guidance following controlled ovarian stimulation approximately three weeks prior. Notably, immediate embryo transfer was not performed. She reported experiencing pelvic pain since the procedure, which initially responded to pain medication but never fully resolved. However, over the past 48 hours, her symptoms significantly worsened, with the pain progressing to involve the upper quadrants of the abdomen.

She had been seen at another facility approximately 12 hours prior to the presentation. At that time, her laboratory results were notable only for an elevated white blood cell (WBC) count of 12.6 K/mL, with 84% neutrophils. An abdominal ultrasound revealed enlarged ovaries with multiple cysts and trace abdominal free fluid, but no other abnormalities were noted (images unavailable).

On examination, her vital signs were significant only for mild tachycardia (heart rate 105-110 beats per minute, regular). She was normotensive (BP 106/77 mmHg) and afebrile (temperature 36.5°C). Her abdomen was distended and diffusely tender to palpation, with guarding and rigidity, findings consistent with peritonitis. Pelvic examination revealed mild cervical motion tenderness without vaginal or cervical discharge. A urine pregnancy test was negative. Laboratory results obtained on admission to our facility are shown in Table 1. Given the absence of a clear source of infection on imaging and clinical signs of abdominal infection, the patient was admitted for intravenous broad-spectrum antibiotics, fluid resuscitation, and observation. She was started on ceftriaxone and metronidazole.

**Table 1:** Pertinent laboratory results and trends

Lab Parameter	Patient Result			Reference Range
	Day of admission	Hospital day 2	Hospital day 3 (pre-operation)	
White blood cell (WBC) (k/mL)	18.6	13.6	11.5	4.0-11.0 k/mL
Neutrophils (%)	91.4	91.5	86.5	40-75%
Hemoglobin (g/dL)	13.8	12.1	10.4	11.5-18.0 g/dL
Hematocrit (%)	40.2	34.7	29.2	36-54%
Platelets (k/mL)	226	148	114	150-400 k/mL
Sodium (mmol/L)	137	-	-	137-145 mmol/L
Potassium (mmol/L)	4.1	-	-	3.5-5.0 mmol/L
Chloride (mmol/L)	114.5	-	-	98-110 mmol/L
Blood urea nitrogen (BUN) (mg/dL)	6.98	-	-	7-17 mg/dL
Creatinine (mg/dL)	0.56	-	-	0.57-1.11 mg/dL
C-reactive protein (CRP) (mg/L)	367.24	-	-	0.2-3.0 mg/L

**Figure 1:**
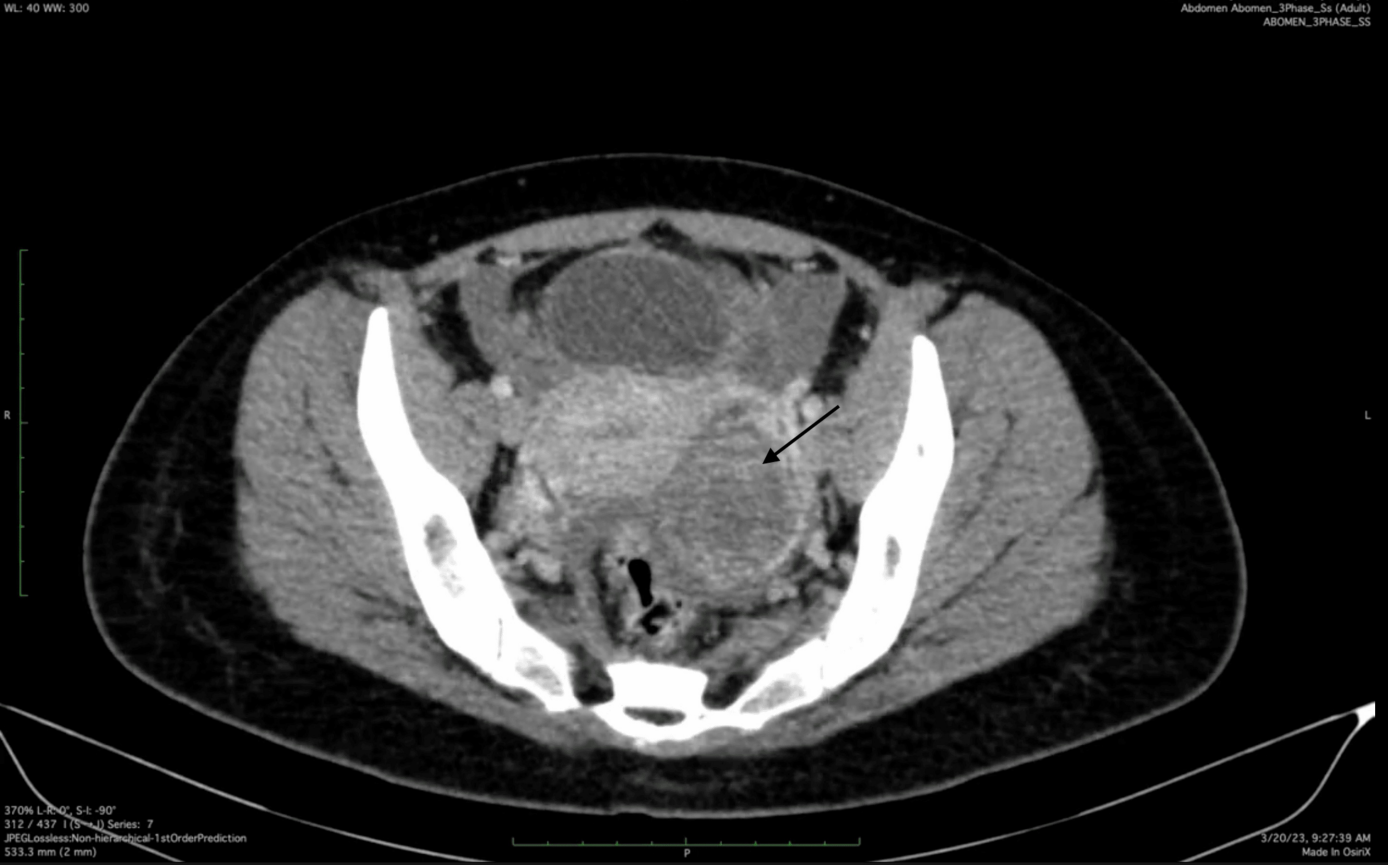
Computed tomography (CT) image taken on hospital day 3, showing an adnexal cyst with peripheral ring enhancement (arrow), suspicious for a tubo-ovarian abscess

On her second day of admission, there was no improvement in her abdominal pain or tachycardia. However, she maintained adequate urine output, and her WBC count had trended downward. Notably, she developed a diffuse urticarial rash, likely related to cephalosporin exposure. A computed tomography (CT) scan of the abdomen and pelvis was obtained, revealing enlarged ovaries with unilocular cysts and minimal pelvic free fluid (Figure 1). There were no signs of appendicitis, pyelonephritis, abscess, or bowel perforation. The patient was subsequently transitioned to intravenous moxifloxacin for 24 hours.

However, no clinical improvement was observed after 48 hours of antibiotic therapy, despite a continued downward trend in WBC count. Therefore, on hospital day 3, a decision was made to perform a diagnostic laparoscopy and abdominal washout in conjunction with general surgery. Intraoperatively, frank pus was noted in all quadrants of the abdomen, with approximately 150 mL aspirated and sent for culture and sensitivity. The abdominal viscera appeared grossly normal, with no identifiable source of infection or abscess cavity. The rectouterine pouch and ovaries showed mild inflammatory changes, but no discrete abscess was identified. Given the absence of a clear source, a prophylactic appendectomy was performed. The abdomen was irrigated with sterile saline, and a drain was placed.

Postoperatively, the patient showed marked clinical improvement, including resolution of abdominal pain, improved appetite, and normalization of heart rate. She was discharged on postoperative day 1 with oral moxifloxacin. At follow-up three days later, the drain was removed after minimal serosanguinous output was noted. Pus culture grew Pseudomonas aeruginosa, which was sensitive to fluoroquinolones but resistant to piperacillin/tazobactam. Pathology of the appendix revealed fibrinous exudates on an otherwise normal specimen. At a subsequent follow-up on postoperative day 10, the patient reported near-complete resolution of abdominal pain, with no complications noted.

## Discussion

The Centers for Disease Control and Prevention (CDC) estimates that 2.4% of pregnancies in the US today are the result of assisted reproductive technology (ART) [1], representing a three-fold increase since 1996. To put this in perspective, according to data from the Society for Assisted Reproductive Technology (SART), the number of women undergoing egg freezing in the US increased from 475 in 2009 to more than 17,000 in 2020. Multiple factors contribute to the rising rate of ART, including the later average age of childbearing and an increased interest in oocyte cryopreservation [2]. Currently, it is estimated that 1 in 8 couples in the US experience infertility [3]. Oocyte retrieval itself involves controlled ovarian stimulation and triggering, followed by egg retrieval via needle aspiration of the follicular cysts, usually under ultrasound guidance and sedation or anesthesia. This procedure is typically performed transvaginally, though a transabdominal approach may be used, particularly in cases of distorted anatomy.

Though considered a safe, low-risk procedure, known complications include vaginal bleeding, intra-abdominal bleeding, intra-abdominal organ injury, ovarian hyperstimulation syndrome, and even adnexal torsion and vertebral osteomyelitis [4,5]. Pelvic infection remains a rare but serious complication of transvaginal oocyte retrieval, with a reported incidence of less than 1% in multiple large series [6-8]. The most likely mechanism by which infection occurs is the transvaginal passage of the needle, which can introduce vaginal microbiota into the sterile peritoneal cavity. Another possibility is inadvertent puncture of the bowel. Risk factors for pelvic infection include a history of prior pelvic inflammatory disease, adnexal adhesions, or the presence of endometriomas [9-12]. Infections are typically polymicrobial [13] and severe, warranting the use of broad-spectrum antibiotics. In our case, cultures grew Pseudomonas aeruginosa, which is a unique finding for this condition. Pseudomonas species have only been reported in a handful of pelvic inflammatory disease cases, typically associated with an intrauterine device [14,15], which was not present in our patient. Initially, the patient did not respond to standard antibiotic therapy for pelvic inflammatory disease; however, once the antibiotic coverage was broadened to include Pseudomonas, the patient showed significant clinical improvement.

Given the low risk of infection, the American College of Obstetricians & Gynecologists (ACOG) recommends against routine antibiotic prophylaxis [16]. However, aside from the immediate morbidity and mortality risks associated with sepsis, patients who develop pelvic infections after oocyte retrieval are at an increased risk of implantation failure [17]. Those who do progress to pregnancy have a higher risk of adverse pregnancy outcomes, such as pregnancy loss, sepsis, and spontaneous preterm birth [18]. Due to these potential complications, clinicians must remain vigilant and maintain a high index of suspicion in patients presenting with symptoms of pelvic inflammatory disease after oocyte retrieval. Once suspected, we recommend a low threshold for initiating antibiotic therapy. Imaging is necessary to rule out abscesses or other sources of infection, and blood cultures should be obtained in severe cases. If antibiotic therapy fails after 24-48 hours, interventional procedures or surgical management should be considered.

## Conclusions

Clinicians must maintain a high index of suspicion for infection in patients presenting with abnormal symptoms after egg retrieval, and a low threshold for initiating broad-spectrum antibiotic therapy, including coverage for atypical organisms. Timely surgical or interventional radiology management is indicated for non-responders to minimize complications.
